# *Saccharomyces cerevisiae*, une levure de plus en plus impliquée dans les infections urinaires: à propos de 3 cas

**DOI:** 10.11604/pamj.2020.35.63.18041

**Published:** 2020-03-03

**Authors:** Mohamed Zaïd Saoud, Mustapha Rhatous, Samira El Mire, Mohammed Lyagoubi, Sarra Aoufi

**Affiliations:** 1 Laboratoire Central de Parasitologie et de Mycologie, Centre Hospitalier Ibn Sina, Rabat, Maroc; 2 Faculté de Médecine et de Pharmacie, Université Mohammed V de Rabat, Rabat, Maroc

**Keywords:** *Saccharomyces cerevisiae*, infection urinaire, Rabat

## Aux editeurs of Pan African Medical Journal

**Figure 1:**
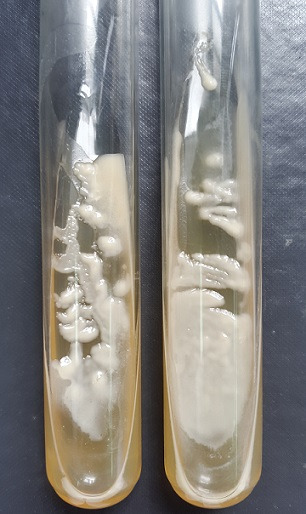
Aspect macroscopique des colonies

**Figure 2:**
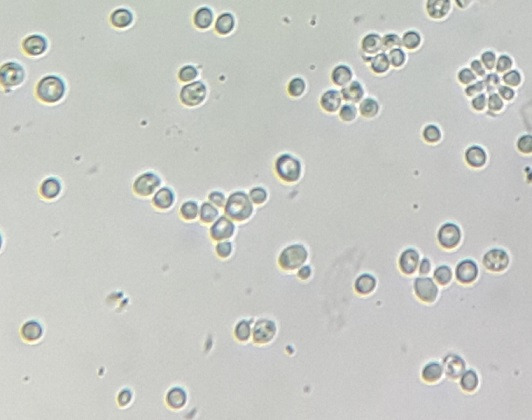
Aspect microscopique des levures après culture (grossissement x 400)

*Saccharomyces cerevisiae* est un champignon ascomycète ubiquitaire. C’est une levure qui est retrouvée chez l’homme sous forme commensale [1]. Elle est largement utilisée dans l’industrie agroalimentaire pour son rôle dans la fabrication de plusieurs aliments, notamment le pain et les boissons fermentées. Elle est aussi utilisée en tant que supplément nutritionnel et en tant que probiotique, notamment les souches de *S. cerevisiae* var. boulardii pour le traitement de divers troubles intestinaux [2]. Longtemps considérée comme inoffensive, cette levure est de plus en plus impliquée dans des cas d’infections parfois invasives, principalement des sepsis fongiques, pouvant être fatales, notamment chez des patients prédisposés [2, 3]. Nous rapportons 3 cas d’infection urinaire à *S. cerevisiae* diagnostiqués au niveau du laboratoire central de parasitologie et de mycologie du centre hospitalier Ibn Sina à Rabat, au Maroc. Les méthodes utilisées pour le diagnostic mycologique sont l’examen microscopique direct du culot de centrifugation des urines, la mise en culture des urines à 37°C sur les différents milieux Sabouraud (simple, additionné au chloramphénicol et additionné au chloramphénicol et à la cycloheximide) et la réalisation d’un auxanogramme (kit AuxaColorTM 2) pour l’identification biochimique des levures isolées. Un cas d’infection urinaire à *S. cerevisiae* a été diagnostiqué en 2015 et 2 cas en 2016. Les trois patientes étaient de sexe féminin. L’âge était compris entre 5 et 40 ans (moyenne d’âge de 26,33 ans). Deux patientes étaient suivies pour diabète sucré, une de type 2 et une de type 1. Une patiente était suivie pour un neuroblastome surrénalien opéré et sous chimiothérapie. Deux patientes, dont l’une était fébrile, ont présenté des signes urinaires à type de brûlures mictionnelles associées à une pollakiurie. Chez les 3 patientes, l’examen microscopique direct des urines a mis en évidence des levures de grande taille. Les cultures sont revenues positives après incubation à 37°C sur les milieux Sabouraud simple et additionné au chloramphénicol et négatives sur le milieu Sabouraud additionné au chloramphénicol et à la cycloheximide. Les colonies étaient blanchâtres et d’aspect lisse et crémeux. L’auxanogramme a permis d’identifier *S. cerevisiae* (Figure 1, Figure 2). Deux patientes ont été mises sous fluconazole avec évolution favorable et une patiente est décédée des suites d’une maladie veino-occlusive probable avant qu’un traitement antifongique n’ait pu être instauré.

*S. cerevisiae* est un champignon ascomycète qui peut être commensal des tractus urogénital, digestif et respiratoire, notamment après un traitement par probiotique [3]. Mais cette espèce, longtemps considérée comme non pathogène, peut aussi être retrouvée sous forme saprophyte dans l’environnement. Il est désormais prouvé que certaines souches de cette levure ont un potentiel pathogène indépendamment du statut immunitaire de l’hôte. Parmi ces souches figurent celles utilisées comme probiotique [2]. Les mécanismes de conversion de la bénignité à la pathogénicité, ainsi que le rôle joué par *S. cerevisiae* dans ces infections, demeurent cependant mal connus [1]. Le premier cas décrit d’infection à *S. cerevisiae* remonte à 1970, chez un patient avec une prothèse de la valve mitrale. Le nombre de cas rapportés augmente les décennies suivantes [4]. Les cas d’infections à *S. cerevisiae* surviennent essentiellement chez des patients présentant des facteurs de risque favorisant la dissémination de la levure: immunodéficience, diabète sucré, corticothérapie, chimiothérapie, néoplasie, dispositif intravasculaire, antibiothérapie, chirurgie abdominale [1, 5]. Les trois patientes de notre étude présentent au moins un facteur de risque d’infection à *S. cerevisiae*: deux sont diabétiques et une est sous chimiothérapie. La voie de contamination des deux patientes diabétiques est probablement à partir d’une colonisation digestive par voie ascendante, mais il existe également des cas de transmission exogène: à travers la peau lésée, lors d’un acte chirurgical ou encore par voie aérienne ou manuportée [5, 6]. La forme clinique la plus fréquente est le sepsis fongique, mais d’autres localisations peuvent être retrouvées [4]. Les infections urinaires sont exceptionnelles. Pillai *et al.* rapportent un cas original de pyélonéphrite aiguë à *S. cerevisiae* chez une femme diabétique souffrant d’insuffisance rénale chronique [1]. Attribuer une infection urinaire à *S. cerevisiae* n’est pas simple étant donné son commensalisme. La présence de signes urinaires évocateurs d’une infection, ainsi que le contexte général du patient et l’isolement de la levure, permettent de trancher entre infection et colonisation. La pousse de la levure est rapide, le temps de génération étant de 1,25 à 2 h [1]. L’identification peut aussi bien se faire par auxanogramme que par biologie moléculaire [3]. Il n’existe cependant pas d’examen sérologique commercialisé pour *Saccharomyces* [1]. Le traitement des infections à *S. cerevisiae* repose sur l’amphotéricine B, qui reste le traitement de choix, ou la flucytosine. Les azolés comme le fluconazole ont une activité plus variable [2]. Les concentrations minimales inhibitrices de *S. cerevisiae* sont plus élevées que celles de *Candida albicans*, ce qui devrait être pris en compte par les prescripteurs, notamment avec l’administration de plus en plus fréquente des probiotiques [1]. Il est aussi recommandé de retirer un éventuel dispositif intravasculaire ou urinaire, notamment en l’absence de prise de probiotique [6]. Le pronostic des infections à *S. cerevisiae* reste réservé du fait du terrain débilité sur lequel elles se greffent. La mortalité peut aller jusqu’à 50%. Cependant, le décès n’est pas toujours imputable à la levure, mais plus souvent aux maladies sous-jacentes [4]. Dans notre étude, une patiente sur trois est décédée, mais ce décès est probablement dû à une maladie veino-occlusive à la suite d’une greffe de cellules souches hématopoïétiques.

## Conflits d’intérêts

Les auteurs ne déclarent aucun conflit d’intérêts.
